# In Vitro Human Cancer Models for Biomedical Applications

**DOI:** 10.3390/cancers14092284

**Published:** 2022-05-03

**Authors:** Jane Ru Choi, Gül Kozalak, Ighli di Bari, Quratulain Babar, Zahra Niknam, Yousef Rasmi, Kar Wey Yong

**Affiliations:** 1 Life Sciences Centre, University of British Columbia, 2350 Health Sciences Mall, Vancouver, BC V6T 1Z3, Canada; janeruchoi@gmail.com; 2 Faculty of Engineering and Natural Sciences (FENS), Sabanci University, Istanbul 34956, Turkey; gulkozalak@gmail.com; 3 Center of Excellence for Functional Surfaces and Interfaces for Nano-Diagnostics (EFSUN), Sabanci University, Istanbul 34956, Turkey; 4 Dialysis and Transplantation Unit, Department of Emergency and Organ Transplantation-Nephrology, University of Bari, 70124 Bari, Italy; ighli87@gmail.com; 5 Department of Biochemistry, Government College University, Faisalabad 38000, Pakistan; ainniebabarrr@gmail.com; 6 Proteomics Research Center, Shahid Behesti University of Medical Sciences, Tehran 1983969411, Iran; niknam_zahra@yahoo.com; 7 Department of Biochemistry, School of Medicine, Urmia University of Medical Sciences, Urmia 5714783734, Iran; 8 Cellular and Molecular Research Center, Urmia University of Medical Sciences, Urmia 5714783734, Iran; 9 Faculty of Medicine & Dentistry, University of Alberta, Edmonton, AB T6G 2R7, Canada

**Keywords:** in vitro model, human cancers, biomedical applications, therapeutic development, tumor biology, cancer markers

## Abstract

**Simple Summary:**

Cancer is a leading cause of death worldwide. While numerous studies have been conducted on cancer treatment, clinical treatment options for cancers are still limited. To date, animal cancer models for cancer therapeutic studies have faced multiple challenges, including inaccuracy in the representation of human cancers, high cost and ethical concerns. Therefore, lab-grown human cancer models are being developed quickly to fulfill the increasing demand for more relevant models in order to improve knowledge of human cancers and to find novel treatments. This review summarizes the development of lab-grown human cancer models for biomedical applications, including cancer therapeutic development, assessment of human tumor biology and discovery of key cancer markers.

**Abstract:**

Cancer is one of the leading causes of death worldwide, and its incidence is steadily increasing. Although years of research have been conducted on cancer treatment, clinical treatment options for cancers are still limited. Animal cancer models have been widely used for studies of cancer therapeutics, but these models have been associated with many concerns, including inaccuracy in the representation of human cancers, high cost and ethical issues. Therefore, in vitro human cancer models are being developed quickly to fulfill the increasing demand for more relevant models in order to get a better knowledge of human cancers and to find novel treatments. This review summarizes the development of in vitro human cancer models for biomedical applications. We first review the latest development in the field by detailing various types of in vitro human cancer models, including transwell-based models, tumor spheroids, microfluidic tumor-microvascular systems and scaffold-based models. The advantages and limitations of each model, as well as their biomedical applications, are summarized, including therapeutic development, assessment of tumor cell migration, metastasis and invasion and discovery of key cancer markers. Finally, the existing challenges and future perspectives are briefly discussed.

## 1. Introduction

Cancer is known as one of the most life-threatening diseases worldwide [1]. In-depth studies of cancer mechanisms are essential, given that this disease is complex and progressive. Traditionally, animal models have been widely used for studies of cancer therapeutics as they provide a relevant tumor microenvironment (TME) to evaluate drug safety and efficacy [2]. However, problems associated with the accurate representation of human cancers, as well as challenges involving ethical controversies, high cost and difficult handling of animal models, have limited their usefulness [3]. Differences in tumor biology in animal models compared to human models explain why some drugs tested in animals are ineffective in humans [4]. To address these challenges, in vitro human cancer models, including two-dimensional (2D) (e.g., transwell-based model) and three-dimensional (3D) models (e.g., spheroid, microfluidic tumor-microvascular and scaffold-based) have been developed for cancer studies [5]. These models offer simple model design, easy operation and result interpretation, leading to a better understanding of different aspects of cancer, such as tumor growth and proliferation, tumor invasion and drug delivery [6]. Indeed, in vitro cancer models are being developed quickly to fulfill the increasing demand for more sophisticated models in order to get a better view of both cancer biology and cancer therapies.

The existing 2D cancer models have revealed tumor progression, which includes genetic alterations of tumorigenic phenotypes, tumor migration and angiogenesis. For example, 2D transwell-based cancer models have been used to study the effects of angiogenesis on tumor cells [7]. However, studies have shown the absence of key receptors and signaling molecules in these models. They were not able to represent the key characteristics of the TME, rendering them less suitable for drug screening [8]. In contrast, 3D in vitro cancer models resemble tumor behavior and complex multicellular TME, providing a more accurate and reliable platform for studies of disease processes and analysis of drug efficacy [9]. For instance, spheroids form clusters, which mimic the morphology and activities of human solid tumors to better understand tumor activities in the human body [10]. Various types of biomaterials (e.g., natural and synthetic hydrogels) have also been investigated to create scaffold-based cancer models for cancer studies [11]. In addition, previous studies have also developed microfluidic tumor-microvascular models for anticancer drug screening [12]. The 3D models discussed above are able to elucidate the role of different types of cells, extracellular matrix (ECM) components and different stimuli in TME. They are particularly suitable for in-depth studies of oncogenesis-related cellular pathways and transcriptomic profiles to develop better anticancer therapeutic agents.

In view of the advancement of in vitro cancer models, there is a strong need for a timely review on this topic. Here, we discuss the most recent advances in in vitro cancer studies. We first review the latest development in the field by detailing various types of in vitro cancer models, including transwell-based models, spheroids, microfluidic tumor-microvascular systems and scaffold-based models. The advantages and limitations of each model, as well as their biomedical applications, are summarized. Finally, the existing challenges and future perspectives are briefly discussed.

## 2. Type of In Vitro Human Cancer Model

### 2.1. Transwell-Based Model

**Figure 1:**
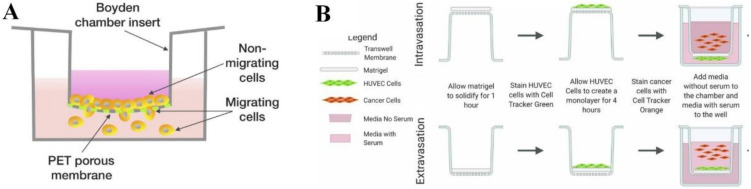
Transwell-based cancer model. (**A**) Basic cell invasion assay. Adapted with permission from [14] © Creative Commons Attribution License (2020). (**B**) Intravasation and extravasation assays. Adapted with permission from [18] © Creative Commons Attribution License (2021).

The transwell-based model is mainly used to assess the migration and/or invasion of tumor cells affected by chemical cues. This culture model consists of two chambers isolated by a porous membrane. The three main variations of transwell-based assays are migration assays, invasion assays and transendothelial migration assays [13]. All the transwell assays are based on the same principle. In this approach, cells are seeded on top of a porous membrane (typically with 8 μm pores) in serum-free media, while serum or chemoattractant-containing media is placed in the bottom chamber (Figure 1A) [14]. Cells that migrate across the porous membrane toward the chemoattractant gradients can be quantified by staining with either a nuclear stain or crystal violet, which is common for counting the migrated cells [13]. For instance, lipopolysaccharide-activated SMMC-7721 cells (a human hepatocarcinoma cell line) were found to have migrated across the porous membrane toward their culture media containing 10% fetal bovine serum (FBS) that was placed in the bottom chamber [15]. Cell invasion assays can also be performed by simply coating the top of the porous membrane with a layer of ECM to mimic the basement membrane of the vasculature [13]. The invasive cells will migrate across the ECM, and the cells can be monitored by fluorometric detection methods or quantified by staining with crystal violet [16]. For example, a study used a transwell-based invasion assay to determine the effect of CD74 on the invasion potential of human pancreatic tumor cell lines. Increased expression of CD74 in the CFPAC-1 and PANC-1 cell lines was found to promote their invasion ability across the Matrigel toward their culture media containing 20% FBS that was placed in the bottom chamber [17].

In transendothelial migration or extravasation assays, a confluent monolayer of endothelial cells is cultured on top of the porous membrane. After ECM production, tumor cells are seeded on the top of this monolayer. The transmigrated tumor cells in the bottom chamber will be collected after 48 h and quantified [19]. This strategy is commonly used to investigate cancer extravasation due to its simplicity and great adjustability. For instance, a study using a transwell-based model showed that CD99 depletion in MDA-MB-231 cells (a human breast tumor cell line) caused a two-fold enhancement of transendothelial migration activity compared to the CD99+ cells [20]. Additionally, an intravasation (movement of cells into blood vessels) assay can also be performed in this transwell-based model by culturing a monolayer of endothelial cells on the lower part of the porous membrane, followed by the seeding of tumor cells in the upper chamber. For example, a study using this intravasation model demonstrated that the c-Met/β1 complex promoted the migration potential of MDA-MB-231 cells to a monolayer of human umbilical vein endothelial cells (Figure 1B) [18]. However, given that this 2D-based model has less physiological association with the real 3D biological systems, more advanced tools are required to mimic the interactions of tumor cells in the TME.

### 2.2. Spheroid or Organoid

In these last few years, several types of 3D culture models have been developed [21]. With the aim of studying tumor biology, especially for anticancer drug screening, spheroid or organoid is one of the most used 3D models. Spheroids reproduce the characteristics of the tumor, such as pathways related to cell signaling, interactions between cell/cell and cell/ECM and gene expression patterns similar to those of the native tumor [22]. Spheroids are structured like spherical cellular units that are usually cultured as free-floating aggregates and presumably of low complexity reflecting tumor organization [23]. Through different cell origins and culture methods, it is possible to obtain several types of spheroids, including multicellular tumor spheroids (MCTS, composed of tumor cell lines and non-adherent support), tissue-derived tumor spheres (composed of tumor cells obtained from enzymatic dissociation of solid tumor), organotypic multicellular spheroids (OMS, composed of tumor and stromal cells from minced solid tumor without enzymatic dissociation) and patient-derived organoids (similar to OMS but requires enzymatic dissociation for long-term culture) [24]. Among the spheroids, MCTS is the most used model for biomedical applications due to its high reproducibility and low cost [25].

**Figure 2:**
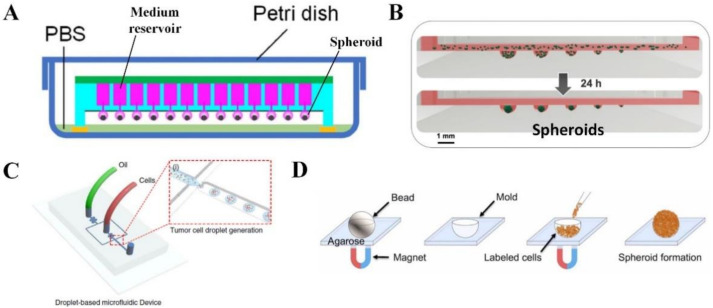
Tumor spheroids. (**A**) A device that allows long-term culture of hanging drop tumor spheroids. Adapted with permission from [30] © Elsevier (2021). (**B**) A multisize hanging drop tumor spheroid array. Adapted with permission from [32] © Creative Commons Attribution License (2021). (**C**) A microfluidic device that traps tumor cells in droplets for formation of tumor spheroids with uniform cell distribution. Adapted with permission from [33] © Creative Commons Attribution License (2021). (**D**) Tumor spheroid formation induced through magnetic levitation. Adapted with permission from [34] © Creative Commons Attribution License (2020).

There are numerous technologies used to generate tumor spheroids that mimic the characteristics of native tumors. One of the cheapest and simplest methods for spheroid formation is the hanging drop technique. Briefly, the device is composed of a lid, a hanging drop well plate, and a tray at the bottom. A cell suspension at the determined concentration was pipetted into each well positioned as part of the hanging portion of the drop plate in the center. It is fundamental to maintain cell hydration by adding water to the peripheral reservoir. The plate is secured with the lid, labeled and incubated at 37 °C in a humidified 5% CO_2_ incubator for 5 days to allow the formation of spheroids. After that, cells were visualized daily to confirm cell aggregation and proliferation. The cultivation medium was changed every other day [26]. This method can be adapted by adding any biological agents in very small amounts, which can be useful in studying the cellular effects of cell-to-cell and cell-to-ECM interactions. In addition, spheroids can be used to co-culture two or more different cell populations to understand the role of the cell/cell or cell/ECM relationship in specific spatial interactions [27]. The conventional hanging drop method successfully produces MCTS from a wide variety of tumor cell lines (e.g., HepG2, MCF-7 and HCT-116) with highly organized tissue-like structures and substantial ECM. The MCTS can be co-cultured with human umbilical vein endothelial cells to generate an early tumor angiogenesis model [28,29]. However, one of the limitations of this method is the short cultivation time. With the aim to address this limitation, an innovative method has been developed, which is the medium integrated superhydrophobic chip for the long-term culture of hanging drop spheroids. The device is structured by two main components: an array of compartments that act as culture medium storage tanks that are connected to each other via a through-hole network, and a patterned superhydrophobic surface containing an array of absorbent dots that anchor cell suspension droplet arrays. Therefore, fresh and large volumes of nutrients can be continuously supplied to the spheroids, enabling long-term culturing (Figure 2A) [30]. This device was successfully used to trigger the spheroid formation of MHCC97-H cells (a human hepatocellular carcinoma cell line) and to culture the spheroids for up to 30 days with high cell viability. Another innovative approach to managing hanging drop spheroids has been developed to overcome several problems, such as reagent cross-contamination, manual pipetting, spheroid preservation and unknown cell culture and analysis time. Polydimethylsiloxane (PDMS)-based drop array chips and pillar array chips use droplet contact-based spheroid transfer technology to allow the repetitive use of spheroids and multiple reagents for various cell analyses, such as viability assessment and immunofluorescent staining. This platform provides less user variation in the operation of the spheroid array and higher spheroid retention [31].

The rotary cell culture system is one of the simplest methods for the large-scale production of spheroids [35]. This system uses a rotating wall vessel bioreactor that creates low shear stress and simulates microgravity for culturing multiple types of cells, allowing them to self-assemble and aggregate to form spheroids [36]. For instance, this bioreactor was used to generate MCTS composed of HCT-116 cells (a human colon carcinoma cell line), human hepatocytes and human mesenchymal stem cells [37]. The cell suspension was rotated in the bioreactor to allow the attachment of cells to hyaluronic acid-based microcarriers that were coated with liver-like ECM, followed by spheroid formation. In the presence of mesenchymal stem cells, an in vitro metastatic colorectal cancer model was established as HCT-116 cells showed active proliferation in the liver spheroid. However, the limitations of the rotary cell culture system for spheroid formation are the variation in spheroid size, and cells could be prone to mechanical damage [35].

Microfluidic spheroid culture is an evolution of conventional hanging drop methods, and this chip-scale technology has a big potential to advance cancer research, especially for drug screening [38], immunotherapy application [39] and studying mechanisms of cancer metastasis [40]. This technology is expanding very fast, relying on the use of small channels with sizes from tens to hundreds of micrometers in height or width for handling small fluid volumes, allowing precise control of the cellular, biophysical and biochemical microenvironment [41]. For instance, a microfluidic device with a multisize microwell (diameters of 300 μm to 1000 μm) was developed to produce MCTSs of different tumor cell lines (liver, lung and colon tumor cells) in different sizes for generating a multisize spheroid array for anticancer drug screening (Figure 2B) [32]. Moreover, fluid volume is reduced in microfluidic 3D culture systems, facilitating the system’s capability as a high throughput screening tool to investigate numerous microenvironmental components that influence tumor development and progression. For example, a 384-micropillar/microwell sandwich 3D cell culture platform was used to develop a human tumor spheroid microarray (pancreatic or breast tumor spheroids) consisting of microencapsulated tumor cells (MiaPaCa-2 or MCF-7) in Matrigel for co-culturing tumor spheroids with natural killer cells for investigating antibody-dependent cell-mediated cytotoxicity [10]. Long-term culturing with cell trapping and in situ MCTS formation can be achieved by a microfluidic chip integrated with U-shaped polyethylene glycol (PEG) hydrogel microstructures with good permeability and biocompatibility [42]. Photolithography was firstly used to fabricate the U-shaped hydrogel microstructures in a pre-assembled microfluidic chip. Trapping of tumor cells (e.g., HepG2 cells) was then carried out by applying gravity against the fluidic flow, and the sizes of spheroids (e.g., hepatocellular carcinoma spheroid) can be structured according to the magnitudes of the U-shaped microstructure. The cells were protected from shear force damage while allowing free diffusion of nutrients, and waste backflow was prevented by the U-shaped microstructures [42]. However, photolithography suffers from a relatively low generation yield, requires expertise and has automation limits. Droplet microfluidics has been used to overcome these limitations by generating spheroids in a quick and efficient manner. To achieve polymerization of the droplet-containing cells, alginate was added to the droplets. The solidified droplets can be cultured in growth medium after polymerization and removal from the oil phase. Separation of the solidified droplets from the oil phase can be easily realized with a further introduction of magnetic beads into the droplets with the help of a magnetic field. Destroying cell clusters within a microfluidic device before forming droplets allows for a more uniform distribution of cells trapped in the droplets for spheroid formation (Figure 2C) [33]. As a result, human glioblastoma spheroids with high cell viability were created using U-87 MG cells through droplet microfluidics.

Magnetic levitation is a novel technique that utilizes negative magnetophoresis to facilitate spheroid formation (Figure 2D) [34]. The spheroids have high sphericity, and their diameter can be easily tuned. Tumor cells were first incubated with magnetic nanoparticles for internalizing the nanoparticles through endocytosis. The magnetically labeled cells were then seeded into an agarose spherical mold, followed by aggregation into a spheroid using an external magnet. This technique successfully created human glioblastoma spheroids using U-87 MG cells. Collagen-embedded spheroid is another strategy that develops a 3D cancer model in conditions that mimic the native TME [43]. Tumor cells were first seeded into an agarose-coated well for spheroid formation. The spheroid was then embedded into a collagen gel. Human bone and breast tumor spheroids with metabolically active cells surrounded by a collagen-based ECM structure were created using U2OS and MDA-MB-231 cells, respectively.

The vasculature has an important role in tumor biology because blood vessels are crucial for maintaining tumor growth. Human tumor cells can be mixed with human lung fibroblasts to form tumor spheroids that can induce the sprouting of human endothelial cells toward the spheroids for vascularization. This was achieved when the tumor spheroids were co-cultured with endothelial cells in a microfluidic chip. Vascularized human brain and breast tumor spheroids were created with this approach using U87-MG cells and MCF-7 cells, respectively [44,45]. In another study, brain tumor spheroids were seeded directly onto a bioprinted blood vessel layer composed of gelatin, alginate, fibrinogen, human lung fibroblasts and human umbilical vein endothelial cells [46]. The blood vessel sprouted toward the spheroids and surrounded them, generating an in vitro brain cancer model with high tumor invasion.

### 2.3. Microfluidic Tumor-Microvascular Model

To better study and understand the kinetics of important cancer progression steps such as angiogenesis, intravasation and extravasation in a controlled microenvironment, a perfusable microfluidic co-culture model of vasculature and tumor cells is required [47]. A microfluidic platform allows the incorporation of multiple cell types and active control components such as micropumps and microvalves for creating multiplex chemical and physical gradients to better recapitulate the complex microenvironment of a tumor. There are three approaches to mimic the blood vessel function in a microfluidic chip, including endothelial cell monolayer, endothelial cell tube and functional vascular network [48]. The endothelial cell monolayer approach is effective in applications, such as the testing of drugs that prevent tumor cell migration, where the tubular geometry of blood vessels is not essential [49,50,51,52]. A study fabricated a 3D microfluidic cell array consisting of three PDMS layers where the bottom layer has microchambers with tumor cells embedded in the PuraMatrix hydrogel, the middle layer is a permeable membrane and the upper layer has microchannels with a monolayer of human dermal blood microvascular endothelial cells [50]. Nutrient supply and waste removal for the encapsulated cells were maintained through a continuous flow of fresh medium in the microchannels. This approach successfully created a human breast cancer model and lung cancer model using T47D cells and PC9 cells, respectively, for high throughput screening for anticancer drugs.

**Figure 3:**
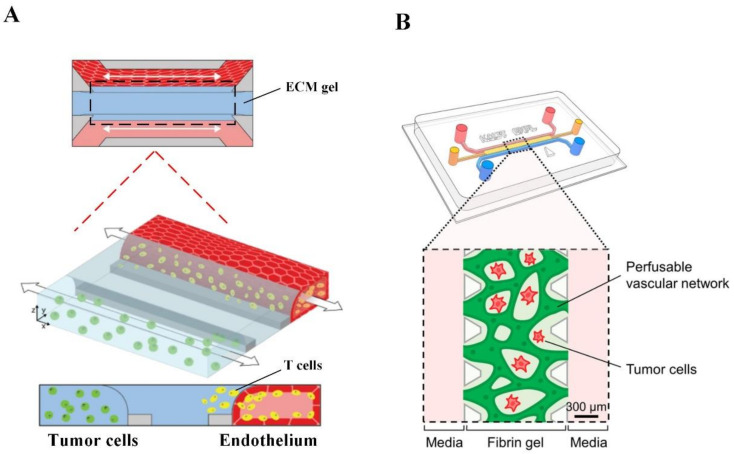
Microfluidic tumor-microvascular model. (**A**) OrganoPlate tumor microvascular models with high throughput screening capabilities. Adapted with permission from [55] © Creative Commons Attribution License (2021). (**B**) A microfluidic chip with functional, cross-linked tumor microvascular networks. Adapted with permission from [57] © ACS Publications (2021).

Some complex microfluidic devices incorporate the tubular structure of blood vessels for studying tumor biology (Figure 3A) [53,54,55]. For instance, one of the OrganoPlates has 40 independent microfluidic chips, and each chip contains two perfusion channels and one in-gel culture channel. This plate was used to create a human in vitro melanoma model using the A375 cell line (Figure 3A) [55]. In this model, human dermal microvascular endothelial cells formed endothelial cell tubes with a good barrier function by growing a monolayer to fully cover the whole inner wall surface of one perfusion channel while A375 cells were grown in the culture channel. Adding T cells activated with different conditions into the lumen of different endothelial cell tubes allows the evaluation of T cell extravasation and migration across the ECM gel channel (indicated by the black dotted frame in Figure 3A) towards tumor cells in a high throughput manner for immunotherapeutic development. Some microfluidic devices allow endothelial cells to sprout in the hydrogel, forming an irregular, cross-linked microvascular network to recapitulate native capillary vessel networks. This is essential for studies that require the cross-linked microvascular network or studies that involve angiogenesis inhibitors [48,56,57]. For example, a study constructed a perfusable, functional liver tumor microvascular network consisting of HepG2 cells and human umbilical vein endothelial cells embedded in a fibrin hydrogel within a microfluidic chip (Figure 3B) [57].

### 2.4. Scaffold-Based Model

Scaffolds are most commonly used to mimic the ECM on which tumor cells can aggregate, grow and migrate. In such 3D cell culture platforms, cells are encapsulated in a polymer matrix composite, and the chemical and physical characteristics of the scaffold material will impress cell properties and response to chemotherapeutics, immunotherapy, radiation or radiochemotherapy [58]. Scaffolds can be made of biological origin or synthetically engineered to mimic the target microenvironment features such as stiffness, porosity and biocompatibility.

#### 2.4.1. Conventional Scaffolds

##### Hydrogels

Hydrogels are 3D networks of cross-linked hydrophilic polymer chains with over 95% water by volume [59]. Hydrogels can be derived from natural or synthetic sources. Natural hydrogels are generally made up of natural polymers such as collagen, Matrigel, hyaluronic acid (HA), alginate, gelatin, chitosan and fibrinogen [60]. These gels, with the existence of multiple endogenous factors, are biocompatible and have natural adhesiveness and the ability to sustain and induce multiple cellular processes, resulting in high cell viability, proliferation and differentiation [61].

Collagen is the most abundant insoluble fibrous protein in the ECM that is widely used in 3D cell cultures [58]. Studies showed that the unusual expression, proteolysis and structure of collagen protein could affect the functions of tumor cells, such as proliferation, initiation, invasion, metastasis and response to therapeutic options [62]. A 3D collagen scaffold has been used to model the 3D glioma cell microenvironment [63]. The study showed that the expression of genes associated with stemness, cell cycle, apoptosis, epithelia-mesenchymal transition, migration and invasion in glioma cell lines, including U87, U251 and HS683, were upregulated in the 3D collagen scaffold compared to the monolayer culture. They concluded that the 3D collagen scaffold increased the malignancy of glioma cells, making it a potential in vitro model for studies on glioma. Matrigel, a basement-membrane matrix secreted from Engelbreth–Holm–Swarm mouse sarcoma cells, has a gelatinous mixture of growth factors and proteins that provides a good mimic of in vivo ECM [58]. Anguiano et al. prepared a hybrid Matrigel-collagen scaffold that mimics the TME [64]. Incorporation of Matrigel into collagen enhanced hydrogel stiffness and induced expression of β1 integrin and metalloproteinase activity in H1299 lung cancer cells. The low concentration of Matrigel in hybrid matrices stimulates efficient tumor cell migration due to strong traction forces exerted by a large number of small-sized focal adhesions. However, in pure collagen scaffolds, cell migration is reduced, likely because of the decreased pore size as well as due to a lower number of focal adhesions and weaker traction forces to maintain an efficient migration. Another well-characterised natural hydrogel is an HA that is expressed at high levels in the TME. HA induces tumor progression and resistance to anticancer drugs. Turtoi et al. fabricated a HA/poly (methylvinylether-alt-maleic acid) (HA3P50) scaffold that promoted proliferation of HepG2 cells and the formation of large cellular aggregates [65]. Growing the hepatocellular carcinoma cells on the scaffold showed liver-like functions, including controlling the release of hepatocyte-specific biomarkers and the synthesis of cytochrome-P450 (CYP)7A1 enzyme, and sensitized the hepatocytes to the anti-tumor effect of cisplatin.

However, natural hydrogels have some drawbacks, such as the limited ability for chemical and physical modifications, a complex and undefined nature, the need to handle low temperatures and poor mechanical properties [59]. For these reasons, synthetic hydrogels have gained traction in recent years due to their high reproducibility and modifiable chemical and physical properties [59,66]. Synthetic hydrogels such as polyvinyl alcohol, poly-2-hydroxyethyl methacrylate and polyethylene glycol (PEG) can mimic biological properties of ECM and TME through functionalization with defined adhesive moieties, encapsulation of growth factors and inclusion of proteolytic sites [58,59]. These materials can also provide mechanical support and stiffness for different types of tumor cells [67]. For instance, a PEG-fibrin scaffold was used to culture A549 cells to create an in vitro model of human lung adenocarcinoma [68]. The cells cultured in a PEG-fibrin scaffold formed tumors with well-organized glandular structures and small luminal structures that resemble the native human lung adenocarcinoma. Additionally, vasculature can be constructed within synthetic hydrogels. For example, endothelial cell-laden alginate microfibers were encapsulated in glioblastoma cell-laden PEG-RGD scaffolds to form vessel-like structures [69]. The glioblastoma cells proliferated faster in the scaffolds that contain endothelial cell-laden alginate microfibers compared to those cultured in the scaffolds with avascular alginate microfibers.

##### Solid Synthetic Scaffolds

Solid synthetic scaffolds provide a physiological context to tumor cell adhesion, proliferation and signaling activities and essential mechanical cues required to retain morphological and genotypic tumorigenicity [70]. These are influenced by the type of materials incorporated into its structure and physical features, such as surface area, pore distribution, pore size and interconnectivity [61]. Various techniques are developed for solid synthetic scaffold production, such as freeze-drying, solvent-casting particulate leaching, electrospinning and 3D printing [60]. Synthetic polymers such as poly (ε-caprolactone) [PCL], poly (glycolic acid), poly (lactic acid) [PLA] and their derivatives are often used in the production of solid synthetic scaffolds to overcome some limitations of natural scaffolds, providing tunable and customizable compositions and mechanical characteristics [70]. For example, an electrospun PCL scaffold was used for HCC1954 cell culturing to fabricate an in vitro model for breast cancer [71]. Electrospun PCL scaffold was found to promote the in vitro formation of tumors identified by the production of mucopolysaccharide and enhanced cancer stem cell population. Additionally, PCL-based tumors showed less sensitivity to doxorubicin and electroporation/bleomycin compared to monolayer cell culture. Overall, electrospun PCL-based tumors may be potential tools for drug screening and preclinical studies. In another study, PLA scaffolds with different morphologies, porosities and pore architectures were produced using the thermally induced phase separation (TIPS) method to culture MDA-MB 231 for creating an in vitro model for breast cancer [72]. They showed that the average pore size of the scaffolds affects the adhesion and morphology of the breast cancer cells. The aggregation of tumor cells and formation of irregular tumor masses were induced efficiently in scaffolds with average pore sizes ranging from 40 to 50 μm. TIPS could be a good technique for finely tuning the solid synthetic scaffold architecture to mimic the TME.

##### Decellularized ECM

**Figure 4:**
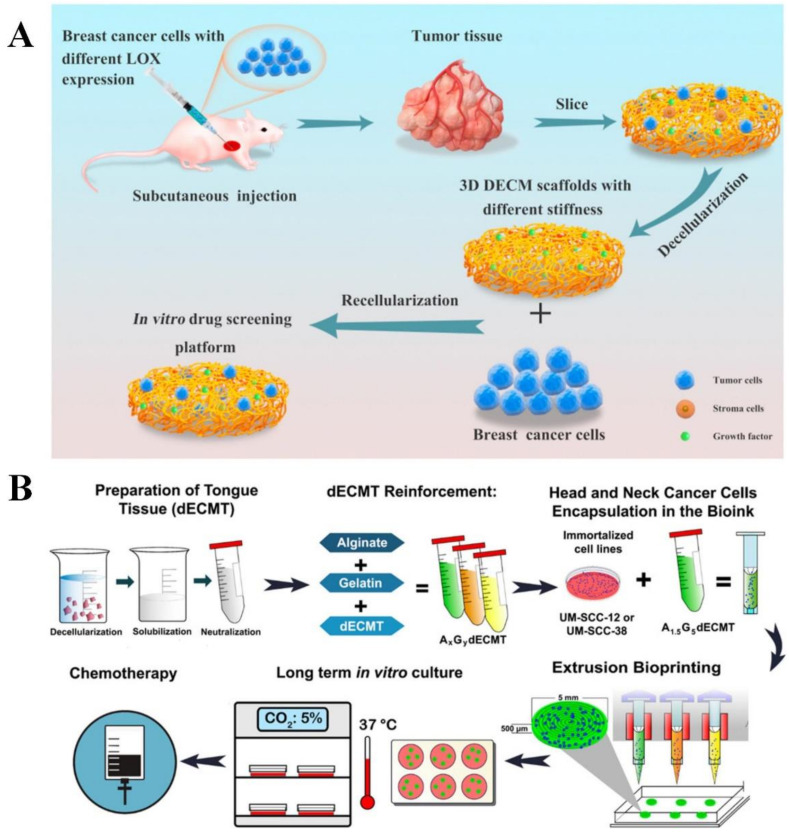
Scaffold-based cancer model. (**A**) Preparation of decellularized extracellular matrix scaffold with different stiffness for in vitro cancer model development. Adapted with permission from [76] © Creative Commons Attribution License (2021). (**B**) Fabrication of a mechanically stable bioprinted scaffold-based cancer model. Adapted with permission from [77] © ACS Publications (2021).

Decellularized ECM (dECM) scaffolds provide native mechanical strength, geometric morphology, flexibility and various matrix components present in the native tissue [61]. The aim of a decellularization process is to eliminate all cellular and nuclear factors for preventing inflammatory responses or immediate rejection after implantation. Recently, various chemical, physical and enzymatic approaches have been reported to prepare dECM scaffolds, including perfusion of the whole organ or tissue using sodium dodecyl sulfate (SDS) and Triton X-100 in a controlled pressure application of supercritical fluid, immersion into the detergent solutions and mechanical agitation. The decellularization method is the most important step because treatment with different detergents and enzymes strongly affects the composition and microstructure of dECM [73]. In addition to the decellularization method, other factors such as dECM sources can affect tumor cell behaviors. For example, the malignant potential of MDA-MB231 breast tumor cells was enhanced in the dECM from adipose stromal cells isolated from obese mice compared to that from lean mice [74]. In another study, patient-derived glioblastoma cells underwent morphological transition and had enhanced migration potential in the physiological relevant patient-derived brain dECM compared to collagen gels [75]. Stiffness of dECM can also influence morphology, migration potential, anticancer drug sensitivity and stemness of tumor cells in vitro. In a study, tumors derived from MDA-MB-231 cells with different lysyl oxidase expression levels were used to prepare dECM scaffolds with different stiffnesses (Figure 4A) [76]. MDA-MB-231 cells cultured in the dECM scaffolds with high stiffness displayed higher expression of drug resistance-associated genes compared to those cultured in the scaffold with low stiffness.

##### Bioprinted Scaffolds

In bioprinting technology, tissue-like constructs are bioprinted using multicomponent bioinks composed of cell-laden fluid materials, matrix components and multiple biomaterials (natural, synthetic or hybrid natural-synthetic biomaterials) [78]. Various biomaterials have been used for bioprinting of different tissue constructs, including collagen, gelatin, chitosan, alginate, fibrin, agarose and PEG [79]. For printing cell-laden constructs, bioinks should have optimal rheological characteristics and viscosity to attain printability and structural stability of bioprinted scaffolds as well as to sustain cell viability during printing and after cross-linking [78]. Three-dimensional bioprinted scaffolds have more advantages compared to other 3D models such as microfluidic tumor-microvascular systems, spheroids and conventional scaffolds because of their ability to precisely control the spatial and temporal distribution of cells and other components in a high-throughput manner and to reproduce the complex architecture of the native tissues [22,79]. Three-dimensional bioprinting provides layer-by-layer precise positioning of living cells and biological materials to produce 3D functional structures [78]. The five widely used 3D bioprinting methods are inkjet-, extrusion-, electrospinning-, stereolithography-based and laser-assisted bioprinting [22].

To date, many efforts have been made to bioprint the TME. Sbrana et al. fabricated a 3D bioprinted tissue construct composed of hydrogels and primary chronic lymphocytic leukemia (CLL) cells to generate an advanced in vitro model for CLL [80]. The leukemic cells survived longer in this construct, showing high viability up to 28 days. Mao et al. fabricated an in vitro liver cancer model consisting of gelatin-alginate-Matrigel and patient-derived intrahepatic cholangiocarcinoma cells by bioprinting [81]. The tumor cells showed invasive, metastatic phenotypes and resistance to anticancer drugs in the 3D bioprinted construct compared to monolayer culture. Another study presented a mechanically stable bioprinted in vitro head and neck cancer model composed of dECM from tongue tissue, alginate, gelatin and UM-SCC-12 or UM-SCC-38 cells (head and neck squamous cell carcinoma cell line) (Figure 4B) [77]. The tumor cells in this construct showed viability above 90% for 21 days and highly expressed cytokeratin, a common marker for head and neck squamous cell carcinoma. This construct was more sensitive to anticancer drugs, including 5-fluorouracil and cisplatin, compared to monolayer culture. Additionally, 3D bioprinting can be used to fabricate vascularized cancer models. In a study, a sacrificial vascular bioink was first embedded in a glioblastoma bioink composed of fibrin, glioblastoma cells, astrocytes and microglia [82]. Following the evacuation of vascular bioink, a mixture of human pericytes and human umbilical vein endothelial cells was injected into the hollow channel to form vasculature. The bioprinted vascularized glioblastoma model showed gene expression profiles that were similar to the native tumors. Three-dimensional bioprinted scaffolds are promising options to prepare an engineered heterogeneous and complex TME for cancer research to achieve better cancer therapeutics. However, there are some limitations to the 3D bioprinting approach, such as lack of reproducibility, standardization of bioprinted constructs, ideal rheological or viscoelastic properties of bioink and intact maintenance of molecular and cellular elements with biocompatible materials [79]. Table 1 summarizes the advantages and limitations of the existing types of in vitro cancer models.

## 3. Biomedical Applications of In Vitro Human Cancer Model

### 3.1. Therapeutic Development for Cancer Therapy

#### 3.1.1. Anticancer Drug

In vitro disease models are utilized for medication development. This approach usually begins with basic research, which aids in the identification of pharmacological targets implicated in illness development. Following that, in vitro disease models may be utilized to screen drug libraries for medicines that affect the pharmacological target of interest. In vitro disease models will be utilized in parallel to analyze, investigate and optimize particular factors such as medication dose in order to predict drug efficacy and toxicity in people. The principle is the same: scientists test a variety of medicines on cells in vitro in order to swiftly exclude those that would not function in mice or people. These models were employed in the development and testing of current anticancer medicines, as well as the creation of novel treatments to substitute animal cancer models in chemotherapeutic testing [84]. In fact, in vitro cancer models are critical in cancer research for investigating genetic, epigenetic and cellular pathways, studying proliferative deregulation, apoptosis and cancer development, defining possible molecular markers and cancer therapies [85,86]. One of the first phases in medication development is generally the evaluation and testing of drugs in tumor cell lines. It permits a huge number of candidate medicines to be tested prior to subscribing to large-scale, costly in vivo clinical studies. Characterizing tumor cell lines in terms of their anchorage independence (soft agarose assay) is also imperative since it can be utilized to figure out which genes and pathways are involved in metastasis as well as their metastatic migration potential and invasiveness capacity [87,88]. Molecular profiling of cell lines that reveals changes in cell cycle regulators and other molecules is essential, enabling anticancer medicines to target cell cycle abnormalities [88]. For example, Hakozaki et al. reported upregulation of the epidermal growth factor receptor and cyclooxygenase-2 genes in a tumor cell line (FPS-1) acquired from an undifferentiated pleomorphic sarcoma (UPS), indicating that this cell line could be used to develop drugs that target these genes or cellular pathways [89]. When Fang et al. characterized cell lines acquired from patients with metastatic and recurrent malignant peripheral nerve sheath tumors, they discovered genes linked to metastatic potential, indicating that these genes could be targeted by therapeutic approaches [90]. Finally, DNA, RNA, proteins, chromosomal and functional profiling were performed on a panel of 60 distinct kinds of human tumor cell lines (NCI60) designed for the generation of anticancer medicines, allowing for an improved clinical translation of the findings of anticancer drug testing [91]. The molecular profiling of tumor cell lines also allows for a more accurate evaluation of cancer types and subtypes, as well as determining which cell lines are most suited for certain studies, improving the screening and research of anticancer medicines [92].

Tumor spheroids are commonly employed to evaluate tumor sensitivity and response to chemotherapeutics, including combination treatments (e.g., chemotherapeutics and small-molecule inhibitors), targeted chemotherapy and drug delivery vehicles [13]. Spheroids are frequently utilized as a high-throughput technique for both negative and positive drug candidate screening in novel drug development [93]. According to studies, gene expression patterns and responses to treatments in tumor spheroid models are more comparable to those in the native tumors [94]. For instance, liver tumor spheroids exhibited drug resistance, which was equivalent to that in native tumors [95]. In comparison to tumor cells grown in a monolayer culture, BT-549, BT-474 and T-47D breast tumor cell lines cultivated as spheroids demonstrated higher resistance to paclitaxel and doxorubicin [96]. Resistance to 5-fluorouracil, regorafenib and erlotinib was found to increase when HCT-116, SW-620 and DLD-1 colorectal carcinoma cell lines were cultured as spheroids with or without co-culturing with fibroblasts and endothelial cells [97]. Altogether, tumor spheroid models are better than monolayer cultured cells as tumor spheroids exhibit drug resistance seen in native tumors [98].

Three-dimensional bioprinting technology also allows for the construction of in vitro cancer models for anticancer drug screening. For instance, a 3D bioprinted breast cancer model that demonstrated doxorubicin resistance was used to evaluate the anticancer effect of lysyl oxidase inhibitor. Lysyl oxidate inhibitor was able to enhance doxorubicin sensitivity of the breast cancer model [99]. In another study, a 3D bioprinted vascularized human glioblastoma model was used to assess the therapeutic effects of the anticancer drug temozolomide and angiogenic inhibitor sunitinib. It was found that the combined treatment was better than temozolomide alone and sunitinib alone in reducing the tumor size [46]. Further research in the field of 3D bioprinting will allow the creation of high-efficiency 3D in vitro cancer models to gain an innovative basic understanding about carcinogenesis mechanisms, as well as to more accurately screen potential anticancer drugs and assist individual drug selection [100]. On the other hand, a microfluidic tumor-microvascular model of human liver cancer was used to assess the anticancer effect of Metuzumab. Metuzumab was able to induce antibody-dependent cell-mediated cytotoxic effects on the liver cancer model in the presence of peripheral blood mononuclear cells [101]. In another study, a microfluidic tumor-microvascular model of human glioblastoma was used to evaluate the effect of antioxidants on glioblastoma [102]. It was found that antioxidant catechins were able to reduce reactive oxygen species in the tumor cells, which could decrease vascular endothelial growth factor secretion from the tumor cells to TME for inducing tumor angiogenesis.

#### 3.1.2. Therapeutic Cells

**Figure 5:**
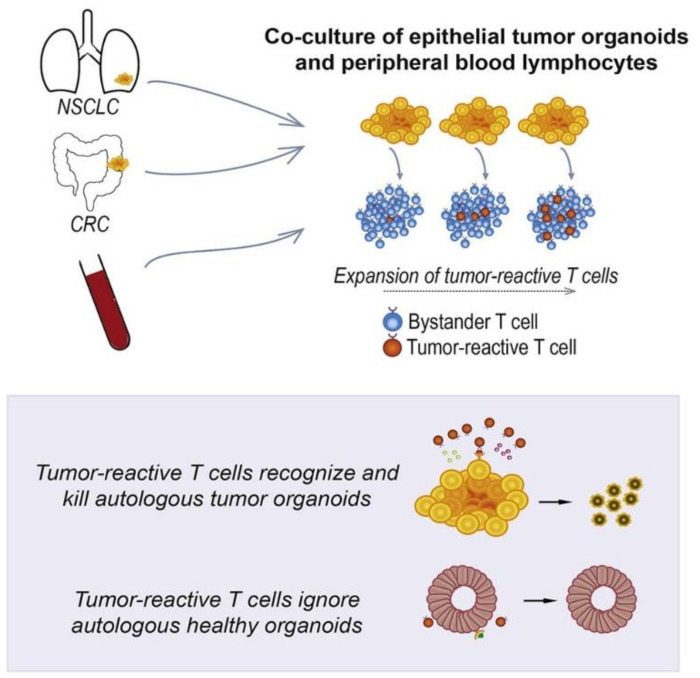
In vitro human cancer models for T cell therapy development. Adapted with permission from [103] © Elsevier (2018).

In vitro tissue models are becoming increasingly important in regenerative medicine, and attempt to replace, restore or regenerate tissue. In the realm of cancer, this has lately taken the form of altering immune cells in vitro so that when they are re-implanted into the patient, they can better fight cancer. For example, chimeric antigen receptor (CAR) T cells were tested on a 3D hydrogel-based in vitro model of human ovarian cancer to assess the anticancer effect of the immune cells before introducing them into patients. CAR-T cells were better than unmodified T cells in mediating the cytotoxic effects on ovarian cancer [84]. Besides CAR-T cells, tumor-reactive T cells can be generated by the co-culture of peripheral blood lymphocytes and tumor spheroids. Peripheral blood lymphocytes co-cultured with human colorectal tumor spheroid and lung tumor spheroid were able to produce CD8+ T cells that kill the colorectal tumor cells and lung tumor cells, respectively (Figure 5) [103]. Immune cells co-cultured with tumor spheroids are a reliable model for assessing the effects of CAR-T and tumor-reactive T cell infusion on cancers, training T cells to identify tumor antigens and predicting patient response to immunotherapy. In general, 3D in vitro cancer models are an important preclinical tool for developing novel immunotherapy tactics for cancer treatment [24].

#### 3.1.3. Phototherapy

**Figure 6:**
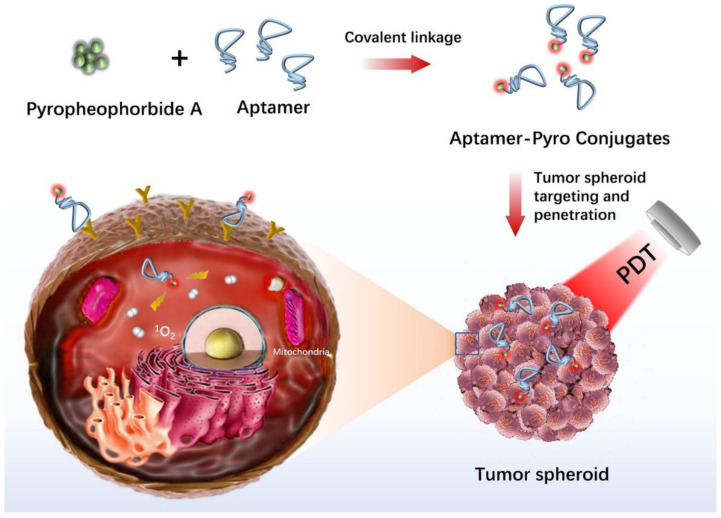
In vitro human cancer models for photodynamic therapy development. Adapted with permission from [109] © ACS Publications (2020).

PDT (photodynamic therapy) is a new theranostic treatment option for a variety of malignancies and illnesses. In the 4T1 cell line (a breast tumor cell line) that is extensively metastatic, Xiaobing et al. investigated the implications of Sinoporphyrin sodium-mediated PDT (DVDMS-PDT) on tumor cell proliferation and metastasis. DVDMS-PDT was cytotoxic to 4T1 cells and able to inhibit the migration of 4T1 cells [104]. Drug resistance is a significant obstacle to cancer therapy. The synergistic impact of drug and phototherapy on the bladder tumor cell line 5637 was examined, and the results showed that blue light irradiation enhances the cytotoxic effect of cisplatin on bladder tumor cells [105]. Analyzing the photosensitizer absorption and penetration through several cell layers has been aided by tumor spheroid culture. For example, the Beckman Laser Institute has employed a human glioma spheroid model to investigate the uptake and localization of 5-aminolevulinic acid/protoporphyrin IX-based regimens for PDT [106]. Xiao et al. investigated the uptake patterns of various porphyrin photosensitizers in a human bladder tumor spheroid model [107]. Hypocrellins and benzoporphyrin derivative monoacid ring A (BPD-MA) were found to penetrate the spheroid deeper than other photosensitizers, including aluminum phthalocyanine, photofrin and protoporphyrin IX. These results suggested that hypocrellins and BPD-MA could be used for bladder cancer phototherapy. In another study, a 3D ovarian tumor spheroid model was utilized to evaluate the phototoxicity of benzophenothiazinium dye EtNBS and its hydroxyl-terminated derivative (EtNBS-OH) [108]. EtNBS was effective in killing the tumor cells in the tumor core, while EtNBS-OH was able to mediate widespread structural degradation of tumors upon irradiation. These two photosensitizers could be used simultaneously for synergistic PDT. Moreover, photosensitizers can be conjugated with aptamers to achieve targeted PDT. For instance, pyropheophorbide, a conjugate with aptamer sgc8 selectively bound to cervical tumor spheroids that overexpressed protein tyrosine kinase 7 and generated singlet oxygen upon red laser irradiation to kill the tumor cells (Figure 6) [109].

### 3.2. Assessment of Tumor Cell Migration, Metastasis and Invasion

One of the most important factors for the progression of metastasis is the vascular system because metastasis of tumor cells mostly occurs within the vascular system. The vasculature of tumors is characterized as unorganized and leaky due to the new formation of vessels that supply nutrients to the tumor. Extravasation and metastasis of tumor cells can easily occur due to the leaky architecture of tumor vessels and secretions of endothelial cells (ECs). Studies in which ECs and tumor cells were co-cultured in both the microfluidic tumor-microvascular system and 3D bioprinted scaffold showed increased tumor cell migration [110,111].

In addition, studies with perivascular cells have shown that they can positively or negatively affect tumor growth and metastasis due to their capacity to stabilize blood vessel structure and permeability [112,113]. Cancer-associated fibroblasts (CAFs) are the main perivascular cells in TME, and their roles in TME are stimulating tumor cell proliferation and facilitating cancer progression by modifying ECM components and metastasis by modulating immune components [114]. In addition, CAFs initiate angiogenesis by supporting ECs that provide nutritional support for tumor growth and development [115]. Furthermore, CAFs positively affect the proliferation and metabolism of tumor cells via autophagy [116]. In a 3D hydrogel-based scaffold, human squamous cell carcinoma cells were unable to migrate independently, but they were shown to invade along CAFs within the matrix [116]. In another study, the requirement for direct cell contact from CAFs to induce tumor invasion was demonstrated in a 3D colon tumor spheroid model [117]. It has been revealed that different CAF signatures are associated with different survival rates of patients with ovarian cancer [118]. Overall, new insights into tumor biology and drug discovery can be gained by examining CAFs and tumor cell interactions in 3D in vitro cancer models.

**Figure 7:**
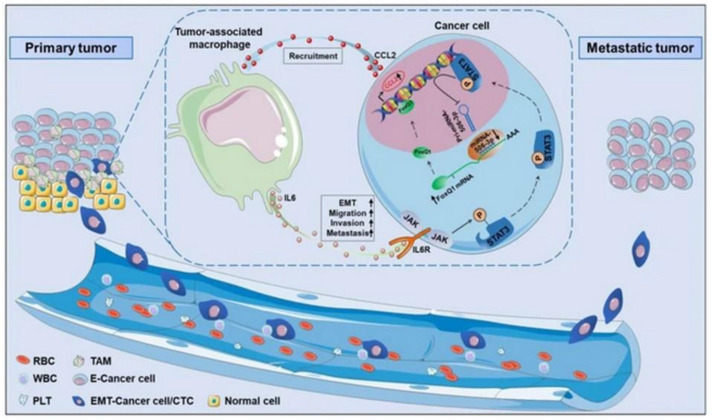
In vitro human cancer models for assessment of tumor biology. Adapted with permission from [123] © Creative Commons Attribution License (2019).

Macrophages are the most abundant immune cells in TME that are derived from monocytes. In general, M1 phenotypes show anticancer properties, while the M2 secrete cytokines and growth factors for promoting inflammation [119]. Results of a study with macrophage/breast tumor spheroid models showed increased cytokines associated with the M2 phenotype, faster oxygen consumption and resistance to cytotoxic drugs [120]. Studying macrophages in 3D in vitro cancer models is essential to better understand the clinical implications of immunotherapeutics. Cancer stem cells (CSC) are a special subpopulation that maintains tumor growth with their self-renewal and differentiation capacity. It provides activation of signaling pathways involved in the cell cycle, growth factor secretion and stemness properties. CAFs were shown to enrich CSCs in lung tumor cells through the de-differentiation process, such as epithelial to mesenchymal transition (EMT) [121]. The EMT process has an important place in the acquisition and maintenance of stem cell-like properties and the invasive phenotype in tumor cells [122]. A study with a colorectal tumor/macrophage transwell model revealed that macrophages produce IL-6, inducing the EMT program to promote tumor invasion, migration and metastasis (Figure 7) [123]. In a 3D bioprinted cervical cancer model, TGF-β was found to induce EMT in HeLa cells [124].

### 3.3. Study of Tumor Biology in Human Cancers Other Than Metastasis and Invasion

Another aspect of tumor biology apart from metastasis and invasion is hypoxia-induced tumor immune evasion and drug resistance. The deprivation of oxygen in tumor mass leads to hypoxia in the tumor. A hypoxic environment enhances the interaction of tumor-immune cells and inhibits anti-tumor immunity [125]. A hypoxic environment creates an invasive and quiescent tumor cell population that could survive stress and escape from the therapeutic effect of the conventional anticancer drugs. Creating 3D in vitro cancer models that exhibit tumor hypoxia and tumor-immune interactions might be more advantageous and therapeutically beneficial over animal models [126]. Therefore, a human tumor spheroid model that recapitulates hypoxic gradients was developed to bridge the gap between classical in vitro cancer studies and animal model studies [127]. Low oxygen tension also promotes glycolysis in tumor cells. This dependence on glycolysis triggers oncogenes and tumor suppressors, defined as the hypoxia response system, allowing tumor cell proliferation, escaping from immune cells and apoptosis [128]. Low oxygen levels can result in an acidic environment. Acidification is a major contributor to tumor invasion and metastasis. Most anticancer drugs are basic, and low pH inhibits the diffusion of chemotherapeutic drugs across the cell membrane [129]. For instance, hypoxic regions in the human head and neck squamous cell carcinoma spheroid model were less sensitive to anticancer drugs, such as evofosfamide, tirapazamine and cisplatin [130].

### 3.4. Discovery of Key Cancer Markers

**Figure 8:**
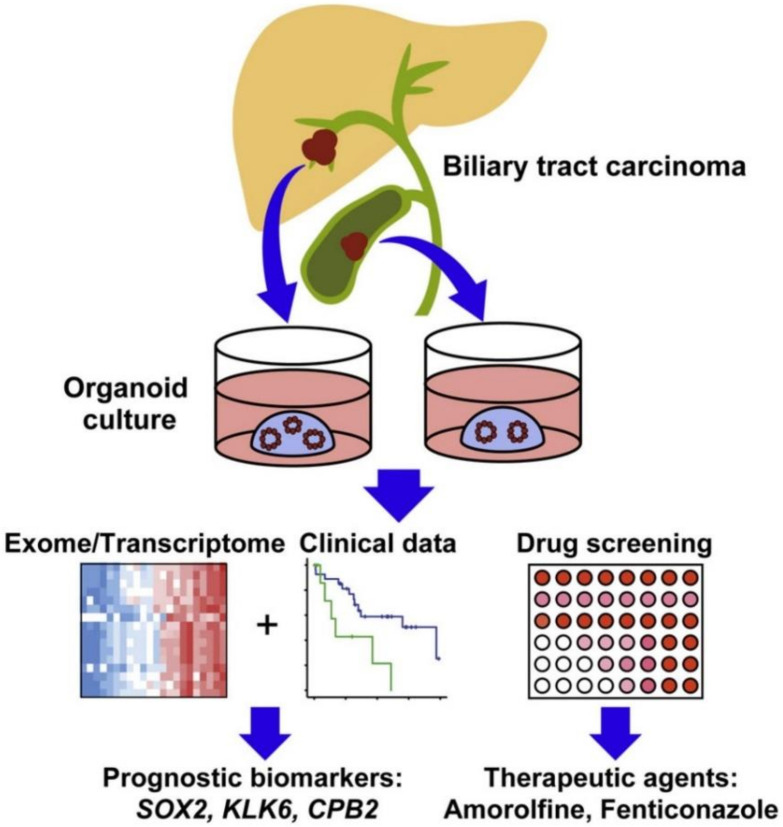
In vitro human cancer models for discovery of key prognostic cancer biomarkers. Adapted with permission from [136] © Elsevier (2019).

Biomarkers that predict cancer are a major requirement used to classify patients and predict their response to cancer therapy. Expressions of some biomarker genes specific to cancer type have been studied in many human tumor spheroids. For instance, extra-domain B of fibronectin was found to be highly expressed in malignant glioma spheroids compared to other tumors, suggesting that it could serve as a potential diagnostic biomarker for malignant gliomas [131]. In another study, two novel glycoforms of prostate-specific antigen (one is a high molecular weight with highly branched N-glycans while another one is low molecular weight without N-glycans) were found to be secreted by prostate tumor spheroids [132]. These biomarkers could improve the sensitivity and precision of screening tests for prostate cancer. Moreover, the response to inhibition of histone methyltransferase EZH2 in colon tumor spheroid models has been associated with the expression of the pro-apoptotic BIK gene [133]. BIK gene expression could be used to predict the patient response to EXH2 inhibition treatment. The resistance of cholangiocarcinoma spheroid models to HSP90 inhibitors appeared to be mediated by the expression of miRNA-21 [134]. miRNA-21 could serve as a potential prognostic biomarker for patients with cholangiocarcinoma. DNA methyltransferase gene expression has been identified as a biomarker of susceptibility to decitabine using breast tumor spheroid models [135]. Genetic profiling of biliary tract carcinoma spheroid models revealed that SOX2 could be a prognostic biomarker for patients with biliary tract cancer (Figure 8) [136]. Cell-free DNA could be collected from the conditioned medium of human pancreatic ductal adenocarcinoma spheroids to obtain mutational profiles of the tumors [137]. This approach could accelerate drug screening on the tumor spheroids derived from patients for facilitating precision medicine in pancreatic cancer.

## 4. Conclusions and Future Perspectives

Cancer is the leading cause of death globally and its incidence is steadily increasing. Although years of research have been conducted on cancer treatment, clinical treatment options for cancers are still limited. In order to find treatments for cancer, the disease itself must be evaluated from every feature, including biological signaling, complex genetics, physical interactions and mechanical characteristics of individual cells [138]. Additionally, several studies have shown that it is not possible to accurately predict drug susceptibility to cancer by using 2D-cultured tumor cells. Moreover, animal models of cancer show differences in tumor biology compared to human pathologies, which explains why promising therapies performed on animal models are often not applicable when tested in humans. As the advent of cancer vaccines, immunotherapy and precision medicine significantly complicate patient care, stronger, patient-specific tools are needed to better inform the understanding and treatment of human cancers. Advances in stem cell biology, cell culture and microfluidics have led to the development of sophisticated bioengineered micro-scale organotypic models that can bridge this gap.

Remodeling of TME, including cell-cell adhesion, tumor-stromal interactions and cell signaling, is necessary for cancer study. It has been revealed that traditional 2D tumor cell culture and animal cancer models are credible in describing the behavior of tumor cells and in interpreting possible mechanism hypotheses. However, full-specified three-dimensional 3D in vitro cancer models that imitate in vivo tumor structures and permit cell-matrix and cell-cell interactions have appeared extraordinarily integral in a variety of diagnostic and therapeutic purposes. In vitro cancer models make this possible for researchers to reprise aspects of the TME using specific cell types, soluble factors and ECM. The study of interactions within the TME and responses to stimuli such as chemotherapy has been performed by controlling various components of the model. So far, several types of cancer models have been introduced, each with its own advantages and disadvantages, and the choice of model for research is chosen according to the inherent differences in the functionality and complexity of different models based on the intended application [13]. The advancement of 3D in vitro cancer models is abridged in terms of modeling design, fabrication method and their potential usage in biology, drug testing and pathogenesis studies. The advancement of modern and complex 3D in vitro cancer model systems utilizing progressed engineering procedures makes better opportunities to discover important mechanisms of cancer and to create modern clinical treatments.

Tumor spheroids reprise the 3D structure and transport phenomena of tumor tissues to study tumor tissue growth and proliferation, invasion into the ECM, immune interactions, drug screening and angiogenesis. The spheroids can recapitulate the basic tumor 3D architecture, including central necrosis, multicellular structures and proliferation gradients, based on the tumor type. Next-generation spheroid tumor models may utilize ECM embedding and co-culturing with other cell types, including immune cells, to illuminate the interaction of immune cells with tumor cells. Transwell-based models are increasingly applied to evaluate the invasion and migration of tumor cells across porous membranes as well as extravasation or intravasation through endothelial monolayers in a simple and high-performance 2D cell culture platform. Next-generation transwell-based models may include patient-specific cells for tumor migration potential analysis.

Microfluidic tumor-microvascular models have been applied to evaluate the tumor microenvironment, including extravasation and migration. Recent advances in the quantification of gene expression in tumor micro-vascular models have been applied to finding the biochemical interactions between tumor cells and blood vessels that evaluate tumor cell proliferation and dormancy and that control tumor-based angiogenesis. Further studies might increase researchers’ perception of the TME and cancer development by manipulating physical signs, like the interstitial flow and shear stress, introduced via the vessel and co-culture of other relevant cell types within the surrounding matrix.

Three-dimensional scaffolds for cell culture have been recently designed to produce the complex and multifactorial environment of native tumors. There is a strong demand for 3D solid tumor scaffolds to improve cell adhesion in cancer biology research. The scaffold-based culture approaches provide physical support, from simple mechanical structures to ECM-like matrices in which cells can proliferate, migrate and aggregate [58]. Combining biomedical engineering knowledge of 3D scaffolding design with knowledge of disease mechanisms, biomarkers and genomic data provides information on the unique design of biomimetic scaffolds that most closely comprise the agents that contribute to the phenotypes of a specific cancer. In vitro 3D tumor models, such as tumor spheroids, are primarily employed for therapeutic monitoring. Tumor spheroids mimic the interactions of cell-cell and cell-matrix in the TME. However, these models do not comprise all aspects of the complex TME, such as the related vasculature and neural networks. Thus, bioprinting techniques can be used to create multicellular, controllable and reproducible tumor models. In all of these applications, the development of biomaterials and the advancement of tissue engineering are constant sources of inspiration for in vitro modeling. Assembly methods, such as controlled spherical formation and bioprinting, dictate how to create sophisticated 3D environments that realistically summarize the metastatic niche.

Apart from these, so far, there is no standard protocol for fabricating the tumor models, which makes it difficult to compare the results among models. This problem will be exacerbated as the scope and complexity of the models available to researchers increase [13]. In addition, the effort toward precision medicine has sparked interest in the adaptation of in vitro cancer models for patient-specific therapies, study of metastatic potential and clinical management. In summary, progress in tumor biology, tissue engineering, 3D cell culture, microfabrication, microfluidics and biomaterials has advanced the development of in vitro cancer models. The advancement of 3D systems of tumor culture bridges the gap between in vitro and in vivo techniques of drug screening as 3D in vitro cancer models extend to evolve better indicators of in vivo drug efficacy. We believe that with new technological advancements, microfluidic tools and other aforementioned models will be developed rapidly for in vitro preclinical cancer studies.

## Figures and Tables

**Table 1:** Advantages and limitations of the existing types of in vitro cancer models.

Type of In Vitro Cancer Model	Advantages	Limitations
Transwell-based [13,14]	Used for studying invasiveness and metastatic potential of tumor cells in a low cost and high throughput manner.	Low physiological relevance. Lack of direct intercellular interactions that are essential for TME.
Tumor spheroid [13,14,83]	Can reproduce 3D architecture of tumors and hypoxic conditions in the spheroid center with direct and paracrine intercellular interactions that are important for TME. Control of uniform spheroid size for standardized drug screening.	Lack of interaction between ECM and cells.
Microfluidic-tumor microvascular system [13,14]	Can reproduce fluid flow, shear stress and chemical gradient profiles that resemble the in vivo conditions. Well-defined vessel endothelium with sizes from capillaries to microvessels and complex networks.	Expensive and requires complicated equipment.
Scaffold-based [14,83]	Resemble the in vivo conditions with complex intercellular interactions and cell-ECM interactions. Bioprinting can precisely control the spatial and temporal distribution of cells and other components such as growth factors.	Expensive for large-scale production. Trouble in cell dissociation from scaffold.
